# Pharmacological maintenance treatment in older age bipolar disorder

**DOI:** 10.1097/YCO.0000000000001101

**Published:** 2026-06-10

**Authors:** Debbie Zittema, Annemieke Dols

**Affiliations:** a Department of Psychiatry, Amsterdam UMC, Vrije Universiteit Amsterdam; b Amsterdam Public Health, Mental Health Research Program; c GGZ InGeest Specialized Mental Healthcare, Amsterdam; d Department of Psychiatry, UMC Utrecht Brain Center, University Medical Center Utrecht, Utrecht University, Utrecht, The Netherlands

**Keywords:** bipolar, older age, maintenance, pharmacological, treatment

## Abstract

**Purpose of review:**

The current review discusses the most recent developments in pharmacological maintenance treatment of older age bipolar disorder.

**Recent findings:**

Older age bipolar disorder (OABD) applies to patients with bipolar disorder aged 50 years and over, a threshold reflecting the association between bipolar disorder and premature aging. Although medication effective in younger age bipolar disorder (YABD) are generally assumed to remain effective in older adults, the disease course becomes more complex in time and age-related changes in pharmacokinetics and pharmacodynamics require careful consideration. Despite the growing aging population, research targeting OABD remains limited. Therefore, the Global Aging and Geriatric Experiments in Bipolar Disorder (GAGE-BD) was initiated by the ISBD (International Society for Bipolar Disorder) OABD taskforce in 2016. Several recent studies on pharmacological maintenance therapy originated from this initiative and are discussed in this narrative review alongside other recent findings. However, no intervention studies have been published in the last 18 months, and the current evidence base largely consists of prospective cohort studies and cross-sectional data.

**Summary:**

When looking at maintenance therapy in OABD, lithium remains first line of treatment but with an adjusted lower therapeutic range. Lithium therapy should not be withheld due to concerns of future kidney function decline.

## INTRODUCTION

Older age bipolar disorder (OABD) refers to patients diagnosed with bipolar disorder aged 50 years and over. This age threshold on OABD has been defined by The International Society for Bipolar Disorders (ISBD) Task Force [1] and reflect the premature aging and reduced life expectancy of approximately 10–20 years [2–4]. Findings from the Global Aging & Geriatric Experiments in Bipolar Disorder (GAGE-BD) project show that bipolar disorder does not fade with age [5^▪▪^]. On contrary, the disease course in OABD often becomes more complex, with longer depressive and manic episodes and shorter inter-episode intervals [6–8]. With ageing, bipolar disorder patients face more frequent physical comorbidities [9] and cognitive deficits [10,11]. A recent study performed in 52 bipolar disorder patients recovering from a manic episode found an inverse relationship between age and insight into illness. However, as it was a small cross-sectional study, it is difficult to relate these findings to the limited and inconclusive existing literature, and should therefore be interpreted with caution [12^▪^]. Social factors like a small social network with lack of social support and loneliness negatively impact stability and quality of life [13–15]. All these above factors collectively make mood stabilization with an effective maintenance treatment strategy in OABD challenging. In addition, changes in pharmacokinetics and dynamics make older patients more vulnerable to side effects and drug interactions. Despite population aging, particularly in Western and East Asian nations, research especially addressing OABD remains limited [16]. The 2018 CANMAT-ISBD guideline has a section that covers pharmacological treatment in OABD [17,18]. They describe that open-label trials, naturalistic studies, and post hoc analyses of mixed aged RCTs suggest that medications efficacious in adults overall will also be effective in older adults, although additional considerations regarding medication tolerability and age-related changes in pharmacokinetics and pharmacodynamics must be taken into account. However, given the above-mentioned factors influencing clinical course and treatment, more research is warranted to present a tailor-made evidence-based treatment regimen for OABD instead of following treatment guidelines for younger age bipolar disorder (YABD). This review focuses on current new insights concerning maintenance treatment of OABD. However, no intervention studies have been published in the last 18 months, and the current evidence base largely consists of prospective cohort studies and cross-sectional data. 

**Box 1:**
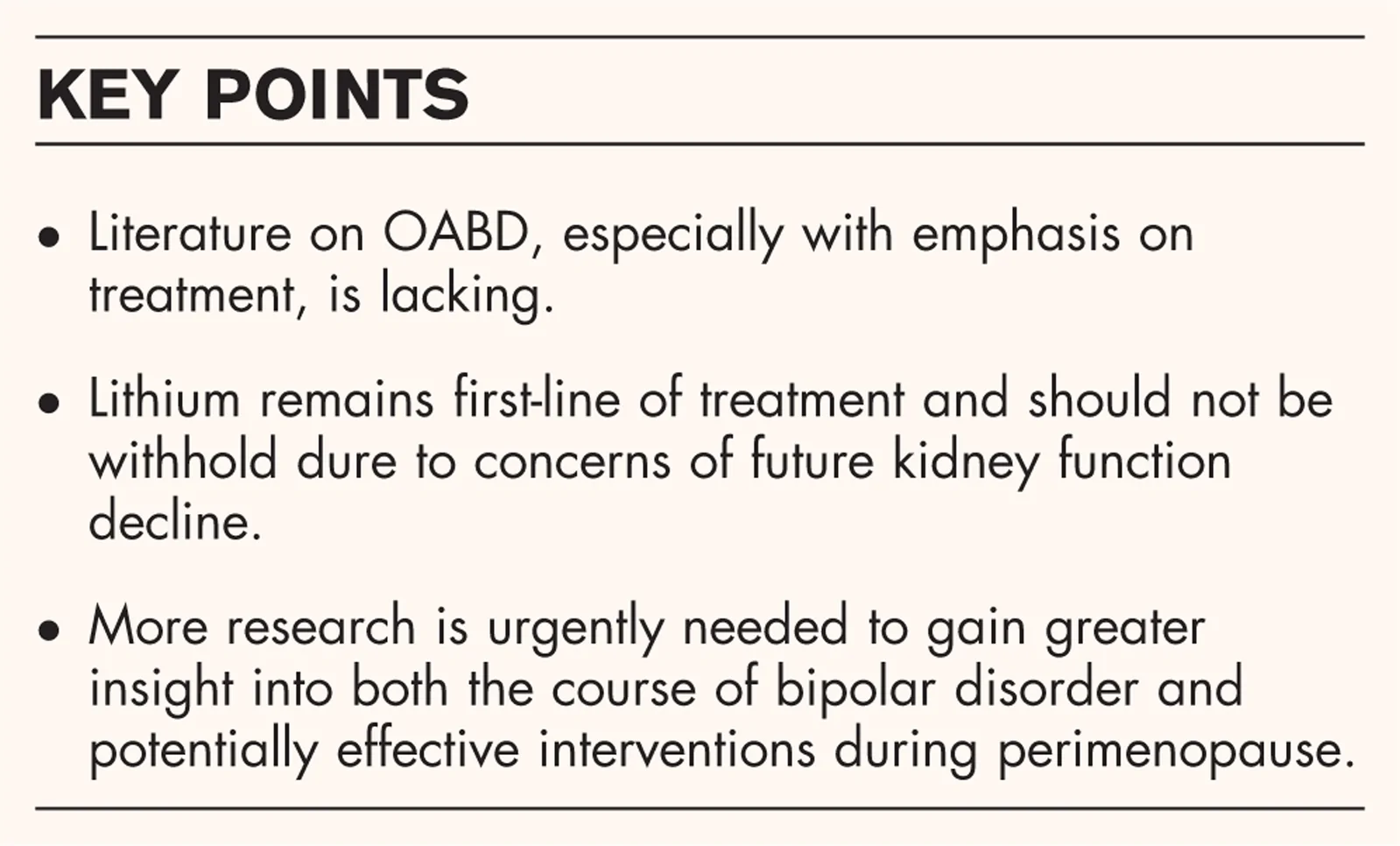
no caption available

## MOOD STABILIZERS

### Lithium

Lithium continues to be a first-line recommendation for treatment in OABD for both mania, depression, and as prophylactic maintenance therapy [19^▪^]. Recently, the position of lithium as preferred choice for maintenance monotherapy in OABD was confirmed in a Delphi study performed by the ISBD task force for OABD with recommendations on lithium use based on the opinion of 25 OABD experts [20]. However, an age-adjusted lower therapeutic range is in order (0.4–0.8 mmol/l for age 60–79 years; 80+ years and 0.4–0.7 mmol/l for age 80+) and should be reported separately by laboratories to enhance proper monitoring by practitioners to minimize renal and other toxicity risks [20]. Routinely lithium measurements used in most clinical practices have sufficient precision within the recommended therapeutic intervals according to a recent analytical precision study [21^▪▪^].

Several studies describe the efficacy of lithium in bipolar disorder in general and in OABD in particular. Lithium seems to have lower discontinuation rates in comparison to valproate (valproic acid or divalproex), quetiapine, olanzapine, venlafaxine, or citalopram [22] and demonstrates slightly superior tolerability and effectiveness in treatment of mania [23]. Results published by the GAGE-BD project, including a recent replication study, showed that OABD lithium users, in comparison to nonusers, had less depressive symptoms, less rapid cycling, fewer psychiatric and cardiovascular comorbidities and better cognitive and overall functioning [24,25^▪▪^]. A recent study performed in 2919 participants (1342 bipolar disorder, 1577 healthy controls) used neuroimaging and machine learning to compare individual's predicted brain age and their chronological age. In this study, bipolar disorder was associated with higher brain-predictive age difference in comparison to healthy controls. However, lithium use was not associated with advanced brain age whereas other mood stabilizers and second-generation antipsychotics (SGA) were [26^▪▪^]. This is in line with growing evidence associating lithium use with a decreased risk of developing neurocognitive disorders [27,28] and in contrast to the belief that lithium may cause neurocognitive disorders. A recent systematic review also concludes that there is no conclusive evidence that long-term lithium treatment is harmful with respect to neurocognitive outcomes in older patients with bipolar disorder [29^▪^]. In contrast to these positive results, another recent study observed no evidence of associations between the duration of lithium use and biological ageing markers, including telomere length, and all-cause mortality. This study used the UK Biobank, an observational study of middle-aged and older adults [30^▪^].

Despite its benefits, physicians seem to be hesitant towards prescribing lithium mostly due to concerns about renal complications. A potential long-term adverse effect of lithium treatment is a decline in kidney function, which may progress to chronic kidney disease and increase the risk of cardiovascular morbidity. However, it is unclear to what extent long-term lithium use leads to significant eGFR decline. Older studies may present a distorted picture of kidney function decline, as treatment in the past was less protocol-driven and higher lithium levels were more common. It is important to understand the risks within current clinical practice. Recently, a Dutch study followed 196 bipolar disorder patients on lithium for 9 years with an average treatment duration of 16 years [31^▪▪^]. Mean age at follow-up was 59 years. They found an average eGFR decline of 0.8 ml/min/1.73 m^2^. Around 41% had an average kidney function decline similar to aging, 48% had a greater kidney function decline higher than can be expected by aging but probably without clinical complications (0.5–2.5 ml/min/1.73 m^2^). Eleven percentage showed a kidney function decline greater than 2.5 ml/min/1.73 m^2^ with a risk of developing chronic kidney disease. Both treatment duration and lithium levels were associated with kidney function decline. Another study in a predominantly Chinese population developed a risk prediction algorithm stratifying 10-year risk of CKD in patients with bipolar disorder undergoing lithium treatment [32^▪▪^]. They found that a model including higher age at bipolar disorder diagnosis together with male sex, more physical comorbidities (especially diabetes and arterial hypertension), higher mean lithium serum levels, fewer use of antipsychotics and mood-stabilizing anticonvulsants, and greater antidepressant exposure significantly predicted CKD development among lithium-users. However, the mean age in this study was 35 ± 13 years.

A more common renal adverse effect of lithium therapy is an impaired urinary concentrating ability affecting 50–70% of patients [33,34] and potentially progressing to nephrogenic diabetes insipidus [33,35], increasing the risk of dehydration [30^▪^,^31^^▪▪^]. This risk is particularly pronounced in older adults due to a diminished thirst response [35,36]. Dehydration, in turn, may precipitate lithium intoxication and contribute to further renal impairment. Assessing urinary concentrating capacity requires laborious tests (e.g. water deprivation or DDAVP test), which are not feasible in psychiatric outpatient practice and symptoms like polyuria and polydipsia are often underreported, especially in OABD [36]. Research into reliable markers of concentrating ability is needed to further improve safety of lithium treatment in older adults.

In conclusion, most patients do not develop chronic kidney disease from lithium, so its use should not be avoided solely over concerns about renal function, especially in healthy older adults without somatic comorbidities. However, impaired urinary concentrating ability, common with lithium, can increase the risk of dehydration and lithium intoxication, particularly in older adults, making adequate hydration important.

### Anticonvulsants

Anticonvulsants are an essential option for maintenance treatment in bipolar disorder but relatively little is known about its use in OABD. Some efficacy data in OABD is available for valproate and lamotrigine [17,18,19^▪^]. The GAGE-BD project recently published an article about anticonvulsant use in almost 2700 OABD patients [37^▪^]. Thirty-four percentage of patients used anticonvulsants. Valproate was the most prescribed anticonvulsant (46%), similar to YABD, followed by lamotrigine (35%) and carbamazepine (13%). Lithium use was significantly lower in anticonvulsant users compared to nonusers. It is possible that these results reflect hesitancy to prescribe lithium in older adults because in younger patients, anticonvulsant use was associated with more lithium use [37^▪^]. Alternatively, the OABD group that used both an anticonvulsant and lithium reflected a lithium treatment-resistant group. The study also demonstrates that anticonvulsant users (compared to nonusers) more commonly use antidepressants, suggesting that anticonvulsants are used in more atypical forms of bipolar disorder or that multiple medication classes are needed. Anticonvulsant users also had more rapid cycling and a higher amount of mood episodes, which suggests that anticonvulsants are again used in the more atypical forms of bipolar disorder. In this study, in comparison to nonusers, cardiovascular comorbidities were more prevalent in anticonvulsant users [37^▪^]. Possibly, physicians perceive anticonvulsant use to be safer for OABD patients with cardiovascular comorbidities or anticonvulsant use increases cardiovascular risk. The latter is supported with studies that suggests that anticonvulsants have an effect on cytochrome P450 enzymes resulting in metabolic changes [38–40], which may cause endocrine side effects [41,42].

## ANTIPSYCHOTICS

Antipsychotics are very commonly used in OABD both as monotherapy and as adjunct to mood stabilizers [17,19^▪^]. In a recent naturalistic retrospective cohort study with 166 older adults treated with lithium in the Netherlands, almost 35% used antipsychotics [26% second generation (SGA) and 9% first generation] as comedication next to lithium [43^▪^]. Evidence on the relative efficacy of specific antipsychotics in OABD is limited. Its use warrants caution. Older adults are more susceptible to drug-induced extrapyramidal symptoms (EPS) typically seen in first-generation antipsychotics. SGA are less prone to cause these movement disorders, although EPS can still occur. However, they more commonly exhibit anticholinergic properties [44,45] that may contribute to cognitive impairment and delirium spectrum disorders (DSD), especially in older adults. OABD patients are at risk of DSD due to impaired cognitive performance, physical comorbidity and other markers of frailty [4,46]. A recent case-crossover study based on the French nationwide claims database confirmed this risk. They reviewed DSD diagnoses in 1.5 million patients with schizophrenia spectrum disorder and bipolar disorder and found a significant association between high anticholinergic burden and in-hospital DSD diagnoses in patients ages more than 60 years [47^▪▪^]. Furthermore, patients are at risk of cardiovascular morbidities when using SGA. Patients with bipolar disorder have a significantly higher rate of mortality due to cardiovascular disease in comparison to the general population. Multiple factors are likely to influence the link between bipolar disorder and cardiovascular disease like low levels of physical activity, poor diet, and smoking. However, SGA use seems to be an important factor with weight gain as common side effect leading to metabolic syndrome [48]. A discontinuation study showed in a subgroup of older patients treated with SGA, in comparison to patients treated with mood stabilizers, higher treatment failure and early discontinuation, more psychiatric hospitalizations and higher mortality rate [49]. Therefore, any potential benefit from antipsychotics must be balanced against the risk associated with their use. Given the limited efficacy data and heightened risk of side-effects, mood stabilizers rather than SGAs appear to be treatment of choice in OABD [50].

## ANTIDEPRESSANTS

Antidepressants use in bipolar disorder remains controversial due to manic switch potential, especially in BD-I. In BD-II, antidepressants may have a more favorable risk–benefit ratio. Selective serotonin reuptake inhibitors (SSRIs), with lower switch potential, can be considered second-line or third-line treatment option for acute management of bipolar depression and maintenance treatment [17], though no efficacy data is available for OABD. A recent study performed by the GAGE-BD project provided cross-sectional international data on OABD individuals (*n* = 746) and YABD (*n* = 692) regarding antidepressant use [51^▪^]. Antidepressant use was less prevalent in OABD compared to YABD, and associated with female sex, lower education, lack of occupation, type II bipolar disorder, higher depression severity, and lower mania scores. Monotherapy rates among antidepressant users were similar in OABD (18%) and YABD (19%). Data published on a retrospective cohort study with 166 older adults treated with lithium (the same study mentioned in the antipsychotics section) showed that all antidepressants combined were prescribed 31.6% during follow-up [43^▪^], distributed as 9% SSRIs, 11.4% tetracyclic antidepressants (TCAs) and 12.6% other antidepressants, overall slightly lower than 38.5% in a Canadian population-based cross-sectional study of later-life bipolar disorder [52].

## LIVING WITHOUT PROPHYLACTIC MEDICATION

Clinical guidelines position prophylactic or maintenance pharmacotherapy as the cornerstone of treatment of bipolar disorder. However, for example, in the Netherlands, it is estimated that nearly half of individuals with bipolar disorder do not use maintenance medication, despite the very few financial barriers to care. In a Dutch mental health survey and incidence study (NEMESIS) of 2022, 51% received mental healthcare, and only 43% used medication in the 12 months before the assessment [53]. Not much is known about these medication-free individuals, as they do not visit psychiatric treatment facilities. The BOLD study (Bipolarity in Older individuals Living without Drugs) investigates how they manage their mood without medication. Preliminary findings suggest that, compared to OABD individuals with medication (Dutch Older Bipolars or DOBi cohort [54,55]), medication-free individuals appear to be relatively successful in terms of psychosocial functioning, although they do not have a milder clinical course than those on maintenance medication, and show a high prevalence of childhood trauma [56]. A recent study combined two Dutch cohorts to investigate coping styles and personality traits in OABD living without prophylactic medication. In this study, living without prophylactic medication was linked to more active problem solving and passive reaction, and less comforting cognitions among OABD [57^▪^]. These results warrant the question whether, for people with bipolar disorder considering to discontinue medication, psychosocial interventions aimed at improving coping styles may strengthen their self-management. However, further research is needed.

## RELAPSE PREVENTION DURING PERIMENOPAUSE

Perimenopause is a midlife transition state that leads to reproductive senescence in women [58]. Women will transition through perimenopause at an average age of 51.4 years but a relatively wide age range between 40 and 58 years is seen [59]. As OABD applies to women with bipolar disorder aged 50 years and over, the perimenopause is an important topic currently often overlooked in clinical practice. Growing evidence indicates that this period is one of heightened vulnerability although scientific data on this topic is still severely lacking. Both an increase in severity and duration of depression [60,61] and mania [62], a shift towards a rapid-cycling pattern [63,64] and a twofold higher relative risk of new-onset bipolar disorder compared to premenopausal women is described [65]. Maintenance therapy effective before perimenopause may become insufficient, yet guidance on managing mood instability during this period is lacking, and it is unclear whether treatment adjustments are needed after menopausal transition. Given the substantial number of women affected, further research is urgently needed on disease course and interventions during perimenopause.

## CONCLUSION

In conclusion, literature on OABD, especially with emphasis on treatment, is lacking while population aging is increasing. When looking at maintenance therapy in OABD, lithium remains first-line of treatment and should not be withhold due to concerns of future kidney function decline, especially in healthy older adults without somatic comorbidities. When reviewing recent studies, it becomes clear that the ISBD task force on OABD has generated a substantial number of publications and is making a significant contribution to expanding knowledge on OABD. However, much work remains to be done, with greater emphasis needed on the area of perimenopause, a period marked by heightened vulnerability about which we still know far too little.

## Acknowledgements

*The authors thank the researchers of the Dutch Older Bipolar study (DOBi) and the GAGE-BD project for many fruitful collaborations.*

### Financial support and sponsorship

*A.D. was one of the initiators of the GAGE-BD project. This project was supported by the International Society for Bipolar Disorders (ISBD) Bowden Massey Strategic Research Initiative and made possible by logistical support from the ISBD.*

### Conflicts of interest

*A.D. is Principal Investigator of the Dutch Older Bipolar study (DOBi) and one of the initiators of the GAGE-BD project. A.D. is former chair and current member of the ISBD taskforce for OABD. This publication's contents are solely the responsibility of the authors and do not necessarily represent the official views of ISBD. The ISBD is a 401c3 nonprofit organization whose mission is to foster international collaboration in education and research. For more information, visit* www.isbd.org.

## REFERENCES AND RECOMMENDED READING

Papers of particular interest, published within the annual period of review, have been highlighted as:

▪ of special interest
▪▪ of outstanding interest
